# Caesarean section by maternal age group among singleton deliveries and primiparous Japanese women: a secondary analysis of the WHO Global Survey on Maternal and Perinatal Health

**DOI:** 10.1186/s12884-016-0830-2

**Published:** 2016-02-29

**Authors:** Kyoko Yoshioka-Maeda, Erika Ota, Togoobaatar Ganchimeg, Mariko Kuroda, Rintaro Mori

**Affiliations:** Department of Community Health Nursing, School of Nursing, Faculty of Medicine, Tokyo Medical University, Tokyo, Japan; Department of Health Policy, National Center for Child Health and Development, Tokyo, Japan; Faculty of Medicine, University of Tsukuba, Ibaraki, Japan

**Keywords:** Advanced maternal age, Caesarean section, Pregnancy, Risk factors

## Abstract

**Background:**

The rising caesarean section rate is an important public health concern that in turn increases maternal and perinatal risks of adverse effects, unnecessary medical consumption, and inequities in worldwide access. The aim of this study was to investigate caesarean section indications by maternal age group and examine the association between age and caesarean section in primiparous Japanese women with singleton births.

**Methods:**

We analyzed the Japanese data of primiparous women with singleton births from the WHO Global Survey on Maternal and Perinatal Health to compare maternal and neonatal characteristics and outcomes between groups with and without caesarean section. Women were divided into 3 maternal age groups (≤29, 30 to 34 and ≥35 years). We performed multivariable logistic-regression analysis to identify characteristics associated with caesarean section.

**Results:**

Of the 3245 women with singleton births were included in the Japanese data, 610 women (18.8 %) delivered by caesarean section, half of whom (*n* = 305) were nulliparous. We included singleton nulliparous women (1747 deliveries) in our analysis. The maternal age 35 years old was associated with higher risks for all caesarean section (adjusted odds ratio [AOR] 1.89, 95 % CI 1.28–2.78) and emergency antepartum caesarean section (AOR 2.26, 95 % CI 1.49–3.40). Intrapartum caesarean section, which is mainly performed for obstetric indications, was not higher among the older maternal age group.

**Conclusion:**

In Japan, advanced maternal age significantly increased the risk for caesarean section; however, intrapartum caesarean section was not higher risk among the older age group. Management of maternal complications would help to reduce the rate of caesarean sections and associated unnecessary medical consumption.

## Background

Despite a decline in the overall birth rate in many developed countries, delayed childbearing has increased in the last few decades as a result of social, educational and economic factors [1]. Childbearing at 35 years or older is commonly defined as advanced maternal age [2, 3]. Previous studies have shown higher risk of adverse pregnancy outcomes including preterm birth, low birth weight, stillbirth, pre-eclampsia, gestational hypertension, gestational diabetes, and delivery of small- or large-for-gestational-age neonates, and obstetric interventions like caesarean section (CS) and assisted vaginal delivery in women of advanced maternal age [4–9].

The World Health Organization (WHO) recommends that the appropriate rate of CS is not above 15 % [10]. However, data from the Organization for Economic Co-operation and Development (OECD) shows that the average rate of CS was 26 % in 2011, which has doubled over two decades [11]. In Japan, although the number of deliveries has decreased, the rate of CS increased 8 % in 1984, and doubled in 2008 to 23.3 % [12]. Previous studies on pregnancy outcomes in advanced maternal age have shown conflicting findings, although most have suggested an increased risk of CS in older women. These studies, however, featured different settings or small sample sizes, and failed to distinguish between elective and emergency CS, and adjustment for potential confounders [13–15]. To address this gap, we conducted a secondary analysis using Japanese data from the WHO Global Survey on Maternal and Perinatal Health (WHOGS), which included 10 health institutions from Japan. The aim of this study was to investigate CS indications by maternal age group, and examine the association between age and CS in primiparous Japanese women with singleton birth.

## Methods

### Study design and settings

The WHOGS was a multi-country facility-based cross-sectional study conducted between 2004 and 2008 including 24 countries and 373 facilities in Asia, Africa and Latin America [16]. The study methodology of the survey is explained elsewhere in detail [17–19]. In brief, a multi-stage stratified sampling design was used to select the sample of countries and health facilities across the world. Twenty-four countries were selected from 12 WHO regions, and in each country the capital city and two provinces were sampled randomly. Further, seven health facilities with at least 1000 deliveries per year that were capable of performing CS were randomly selected from each these areas. If there were less than 7 facilities, all facilities in that selected areas.

### Data collection

We compiled data from the WHOGS of 3356 women and their infants collected from 10 facilities in Tokyo, Okayama and Nagano prefectures in 2008. All women giving birth in participating health institutions over a three-month period in 2008 were recruited in this study. Individual and health institution data were collected using standard forms. Individual data was obtained from medical records by trained health staff before hospital discharge or the 8^th^ day postpartum for mothers and the 7^th^ day after birth for neonates, with no identification of individuals involved. Collected data included maternal demographics, history of previous pregnancies, medical conditions and complications during the last pregnancy, mode of delivery and birth outcomes. Health institutional data were obtained by interview with the director or head of obstetrics. The data included were: infra-structure, basic emergency medical and obstetric care, availability of laboratory tests, anesthesiology resources, intensive care units, human resources and training. The study follow-up period was conducted at 8 days postpartum for mothers, and at 7 days after birth for newborns.

### Study sample and variables

In our analysis, we included all primiparous women with singleton deliveries from the 10 participating health institutions in Japan. Women who gave birth for the first time were defined as primiparous. Multiple births, and women who had given birth one or more times, were excluded from the study sample.

The main exposure variable of maternal age was categorised into three groups: ages 20–29, 30–34 and 35 years or older. Outcomes were pregnancy and labour complications, and mode of delivery. The following pregnancy and labour complications were included: maternal hypertension (presence of having pregnancy-induced hypertension, preeclampsia and/or eclampsia), prelabour rupture of membranes, vaginal bleeding in 2^nd^ half of pregnancy, fetal growth impairment, preterm birth (<37 weeks) and low birth weight (<2500 g). Mode of delivery was categorised as spontaneous vaginal delivery, instrumental vaginal delivery and CS. Further CS was categorised as elective CS (defined as CS performed before the onset of labour for medical and obstetric indications or at the request of the mother), emergency antepartum CS (defined as CS performed before onset of labour for medical and obstetric indications) and intrapartum CS (defined as CS performed after the onset of labour for failure to progress, failure of induction of labour, fetal distress or intrapartum hemorraghe). Available indications for CS in the dataset were as follows: pre-/eclampsia, suspected fetal growth impairment, fetal distress, failure to induction, cephalo-pelvic disproportion, breech or malpresentation, and other fetal, maternal and obstetric indications. We created the variable named “having two and more medical indications for CS” for women who presented with two and more indications for CS.

Available individual variables in the dataset were: marital status (married/cohabiting and single), maternal education, number of antenatal care visits, maternal pre-pregnancy body mass index (BMI) (25.0 and >25 kg/m^2^), height (<1.5 m and >1.5 m), pre-existing medical conditions, gestational age at delivery (<37, 37 to 41 and ≥41 weeks) and birth weight (<2500, 2500 to 4000 and ≥4000 g). As maternal education was missing for approximately 25 % of women in the dataset, we excluded this variable from the analysis. A medical condition was defined as the presence of any of the following: chronic hypertension, cardiac/renal diseases, chronic respiratory conditions, diabetes mellitus, sickle cell anaemia, severe anaemia and pyelonephritis or urinary infection.

Type of health institution was categorised by ownership as public or private institutions. WHOGS data from Japan is not publicly available.

### Ethical approval

Preceding this analysis, ethical approval was obtained from ethical review board of the WHO and each hospital.

### Statistical analysis

We used frequencies to examine the study population characteristics, pregnancy and labour complications, and indications for CS among maternal age groups.

The risks of maternal age for all CS, elective CS, emergency antepartum CS and intrapartum CS were explored separately via multivariable logistic regression analysis. For each regression analyses, marital status, maternal height, pre-existing medical conditions, birth weight and type of health institution were considered as confounders. Because of strong correlation between maternal pre-pregnancy and maternal height (correlation coefficient = 0.9), only maternal height included in the regression analyses. The youngest age group (20 to 29 years of age) served as a reference group in the multivariable analyses. All estimates of association we accounted for the WHOGS study design as clustering of women within facilities and facilities with selected areas. The point and variance estimation were obtained by using the first-order Taylor linearization. Health institutions were considered as primary sampling units and areas as strata. Pearson *x*^2^ statistics and multivariable logistic regression analysis were conducted accounting for multistage cluster sampling, using “svy” procedure in STATA 13.1 (Statacorp, College Station, TX, USA). Statistical significance was defined as *p* < 0.05.

## Results

**Fig. 1:**
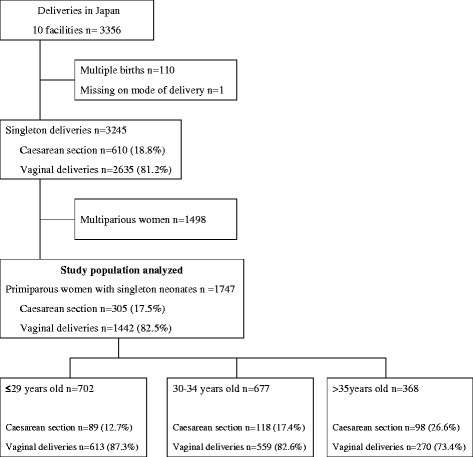
Study flow chat

The WHOGS data included 3356 deliveries from 10 facilities in three prefectures in Japan during the study period. After excluding for multiple births (110 deliveries) and missing data on mode of delivery (1 delivery), 3245 singleton births remained. Of these singleton births, 610 (18.8 %) women delivered by CS, and half of them (*n* = 305) were nulliparous. A total of 1747 nulliparous women with singleton neonates were included in this analysis. The study population consisted of 702 (40.2 %) women aged 29 years or younger, 677 (38.8 %) aged 30–34 years, and 368 (21.0 %) aged 35 years or older (Fig 1).

Table 1 shows the characteristics of the study population. Women aged 35 years or older were more likely to have 12 or more antenatal care visits (47.7 % vs 36.3 % in women aged ≤29 years and 45.4 % in women aged 30–34 years), and a pre-existing medical condition (26.5 % vs 14.0 % in women aged ≤29 years and 15.1 % in women aged 30–34 years). Women aged ≤29 years were less likely to deliver at private institutions (55.6 % vs 64.7 % in women aged 30–34 and >35 years).

**Table 1:** Characteristics of nulliparous women with singleton birth *N* = 1747

Characteristics	Maternal age group, years						Adjusted *x* ^2^, *P*
	≤29		30–34		≥35		
	(*n* = 702)		(*n* = 677)		(*n* = 368)		
	*n*	%	*n*	%	*n*	%	
Marital status							
Maried/cohabited	661	94.4	665	98.5	358	97.3	<0.001
Single	39	5.6	10	1.5	10	2.7	
Number of antenatal care visits							
< =12	440	63.7	361	54.6	186	52.3	<0.05
> 12	251	36.3	300	45.4	170	47.7	
BMI (kg/m^2^)							
18.5 to 25.0	446	63.5	429	63.4	213	57.9	0.19
> 25	256	36.5	248	36.6	155	44.1	
Height							
< 1.5 m	30	4.3	23	3.4	13	3.5	0.44
> 1.5 m	672	95.7	654	96.6	355	96.5	
Pre-exiting medical conditions^a^	98	14.0	102	15.1	94	26.5	<0.001
Health institutional type							
Public	312	44.4	239	35.3	130	35.3	0.23
Private	390	55.6	438	64.7	238	64.7	

^a^Pre-exiting medical conditions was defined as presence of any of followings: chronic hypertension, cardiac/renal diseases, chronic respiratory conditions, diabetes mellitus, sickle cell anaemia, severe anaemia and pyelonephritis or urinary infection

Study-design based Pearson’s chi-2 test was calculated

Table 2 shows pregnancy and labour complications by age group in primiparous women with singleton births. The advanced maternal age group was more likely to have maternal hypertension (12.0 % vs 4.3 % in women aged ≤29 years and 6.1 % in women aged 30–34 years), preterm birth (8.7 % vs 4.5 % in women aged ≤29 years and 5.2 % in women aged 30–34 years), elective CS (10.3 % vs 4.1 % in women aged ≤29 years and 6.8 % in women aged 30–34 years) and emergency antepartum CS (6.5 % vs 2.3 % in women aged ≤29 years and 3.7 % in women aged 30–34 years) than other age groups.

**Table 2:** Pregnancy and labour complication by age groups in primiparous women with singleton births *N* = 1747

	Maternal age group, years						Adjusted *x* ^2^, *P*
	≤29		30–34		≥35		
	(*n* = 702)		(*n* = 677)		(*n* = 368)		
	*n*	%	*n*	%	*n*	%	
Maternal hypertension^a^	30	4.3	41	6.1	44	12.0	<0.001
PROM	171	24.4	157	23.2	90	24.5	0.85
Fetal growth impairment	17	2.4	13	1.9	10	2.7	0.68
Preterm birth (<37 weeks)	32	4.5	35	5.2	32	8.7	<0.05
Low birth weight (<2500 g)	77	10.1	70	10.3	50	13.7	0.11
Instrumental vaginal delivery	53	7.6	59	8.7	43	11.7	0.08
Elective CS	29	4.1	46	6.8	38	10.3	<0.01
Emergency antepartum CS	16	2.3	25	3.7	24	6.5	<0.05
Intrapartum CS	44	6.3	47	6.9	36	9.8	0.20

*CS* caesarean section, *PROM* prelabour rupture of membrane

^a^Maternal hypertension was defined as presence of pregnancy-induced hypertension, preeclampsia and eclampsia

Study-design based Pearson’s chi-2 test was calculated

Table 3 illustrated CS indications by age group in primiparous women with singleton births. The proportion of women who underwent CS for indication of preeclampsia/eclampsia, suspected fetal growth impairment and other obstetric and maternal complications were highest among women aged >35 years. However, these differences were not statistically significant. CS for indication of dystocia and other fetal complications were significantly higher in women aged 30–34 years, whereas a significantly higher proportion of women aged >35 years had two and more indications for CS (42.9 % vs 33.7 % in women aged ≤29 years and 26.3 % in women aged 30–34 years).

**Table 3:** CS indication by age group in primiparious women with singleton births *N* = 305^a^

CS Indications	Maternal age group, years						Adjusted *x* ^2^, *P*
	≤29		30–34		≥35		
	(*n* = 89)		(*n* = 118)		(*n* = 98)		
	*n*	%	*n*	%	*n*	%	
Pre-/eclampsia	3	3.4	10	8.5	13	13.3	0.05
Suspected fetal growth impairment	3	3.4	5	4.2	7	7.1	0.45
Fetal distress	30	33.7	28	23.4	32	32.6	0.22
Dystocia	20	22.5	40	33.9	17	17.4	<0.05
Failed induction	11	12.4	10	8.5	13	13.3	0.49
Breech/malpresentation	31	34.8	29	24.6	29	29.6	0.27
Other maternal medical indications	11	12.4	11	9.3	17	17.4	0.21
Other fetal indications	4	4.5	10	8.5	1	1.0	<0.05
Other obstetric indications	14	15.7	17	14.4	26	26.5	0.05
Having 2 and more indications	30	33.7	31	26.3	42	42.9	<0.05

*CS* caesarean section

^a^Total number of deliveries restricted to women who delivered by caesarean section

Other maternal medical indications included caesarean sections indicated for 3^rd^ trimester vaginal bleeding, suspected/imminent uterine rupture, genital herpes/extensive condyloma and any other maternal medical complications. Other fetal indications included postmortem caesarean section and any other fetal indications. Other obstetric indications defined as conditions such as *placenta previa* without bleeding

Study-design based Pearson’s chi-2 test was calculated

Table 4 showed risk factors for CS in primiparous women with singleton births. Compared to women aged ≤29 years after adjustment for marital status, maternal height, pre-existing maternal medical conditions, maternal hypertension, birth weight and type of health institution, risk of CS were 1.40 (95 % CI 1.02–1.92) and 1.89 (95 % CI 1.28–2.78) times higher in women aged 30–34 years and >35 years, respectively. Women aged >35 years had a significantly higher risk of emergency antepartum CS (AOR 2.26, 95 % CI 1.49–3.40) compared to women aged ≤29 years.

**Table 4:** The association between maternal age and caesarean section in primiparious women with singleton births. Results of multivariable logistic regression analyses

	Maternal age, years								
	≤29	30–34				≥35			
		Crude OR (95 % CI)		Adjusted OR (95 % CI)		Crude OR (95 % CI)		Adjusted OR (95 % CI)	
All CS	ref	1.45	(1.10–1.91)*	1.40	(1.02–1.92)*	2.50	(1.74–3.56)***	1.89	(1.28–2.78)**
Elective CS	ref	1.69	(1.11–2.55)*	1.55	(0.94–2.55)	2.67	(1.54–4.61)**	1.70	(0.88–3.30)
Emergency antepartum CS	ref	1.64	(0.68–3.97)	1.76	(0.73–4.25)	2.99	(1.94–4.61)***	2.26	(1.49–3.40)**
Intrapartum CS	ref	1.11	(0.63–1.98)	1.05	(0.56–1.97)	1.62	(0.97–2.69)	1.44	(0.92–2.25)

Multivariable logistic regression analysis with adjustment study design effect and risk factors. Risk factors were included: marital status, height, pre-exiting medical conditions, maternal hypertension, birthweight and health institutions ownership. Pre-exiting medical conditions was defined as presence of any of followings: chronic hypertension, cardiac/renal diseases, chronic respiratory conditions, diabetes mellitus, sickle cell anaemia, severe anaemia and pyelonephritis or urinary infection. Maternal hypertension was defined as presence of pregnancy-induced hypertension, preeclampsia and eclampsia

*CS* caesarean section, *ref* reference group

* < 0.05 ** < 0.01 *** < 0.001

## Discussion

To our knowledge, this is the first multiple facility-based study with a reasonable sample size focusing on the association between maternal age and CS in Japan. The strength of this secondary analysis was the pretested, standardised data collection form used to obtain individual and health facility risk factors for CS across randomly selected health facilities, therefore the study results could be generalisable to facilities in Japan.

We found that women having their first childbirth and aged >35 years more likely to have maternal hypertension and higher risk of all CS, elective CS and emergency antepartum CS.

After adjustment for potential confounding factors, risk of all CS and emergency antepartum CS were significantly higher in women aged >35 years compared to primiparous women aged ≤29 years with singleton births. The proportion of women having CS indicated for preeclampsia/eclampsia, suspected fetal growth impairment, other obstetric and maternal complications, and having multiple indications were significantly higher in women aged >35 years [20]. Previous research also highlights the effect of advanced maternal age increasing the risk for CS and operative vaginal delivery in the Asian population [21]. However, previous studies have not explored whether elective or emergency CS differs by indication [16, 20–22]. As well as increasing risk for CS, delaying family planning increases the use of assisted reproduction technology, medical costs, and adverse outcomes [3]. To promote safe pregnancy and delivery, the development of supportive health policies and a community healthcare system is needed for younger as well as older mothers.

This study has several important limitations. First, the study findings are not generalisable to the whole country as it includes few facilities out of thousands. Second, this is cross-sectional study and we were not able to identify the cause-effect relationship of CS. However, to the best of our knowledge, it is the first study on maternal age and caesarean section that obtained data from multi-facilities with most available data and indications on caesarean section in Japan.

## Conclusion

In Japan, advanced maternal age significantly increased the risk for CS. However, intrapartum CS—which is generally undertaken for obstetric indications—was not higher risk among the older age group. Management of maternal complications would help to reduce the rate of CS and the associated medical consumption.
